# Application of Kolmogorov–Sinai Metric Entropy to Determine the Exploitation Parameters of Epoxy–Glass Composites with Carbonisate

**DOI:** 10.3390/ma18214858

**Published:** 2025-10-23

**Authors:** Agata Wieczorska, Grzegorz Hajdukiewicz

**Affiliations:** Faculty of Marine Engineering, Gdynia Maritime University, Morska St. 81-87, 81-225 Gdynia, Poland; g.hajdukiewicz@wm.umg.edu.pl

**Keywords:** flexural test, Kolmogorov–Sinai metric entropy, pyrolysis, carbon-based filler, epoxy–glass laminates

## Abstract

This study investigates how the addition of a carbon-based filler obtained through the pyrolysis of medium-density fibreboard (MDF) waste affects the mechanical behaviour of epoxy–glass laminates. Two laminate series with different matrix-to-reinforcement ratios (60/40 and 65/35) were fabricated and modified with carbonised particles of up to 500 μm in size, introduced at 5% and 7.5%. The strength of the samples made of the materials mentioned above was assessed in a static three-point bending test by analysing the values of stresses ($\sigma_{fM}$) and strains ($\varepsilon_{fM}$). For an in-depth analysis of the dynamics of the destruction process, the recorded deformation data were subjected to Kolmogorov–Sinai metric entropy ($E_{K-S}$). The test results showed that the addition of carbonisate in series A (60/40) increased the flexural strength by 32.56% for the sample with 5% addition and by 27.08% for the sample with 7.5% addition, compared to the reference material. In series B (65/35), characterised by a higher resin content, the opposite effect was observed—a decrease in strength of 9.89% (for 5% carbonisate) and 15.53% (for 7.5% carbonisate). The use of $E_{K-S}$ calculations in combination with phase portrait reconstruction to analyse the results obtained allowed for the precise determination of the limit values of stresses and strains ($\sigma_{fMK\_S}$ and $\varepsilon_{fMK\_S}$) at which irreversible structural changes occur in the material, initiating the destruction process. This method proved to be an effective tool for identifying early signs of composite degradation, which is crucial for assessing its long-term strength and designing safe structures.

## 1. Introduction

Recent studies have shown that the geometry and internal structure of composite laminates have a significant impact on their behaviour in bending and compression tests, even when the material composition remains unchanged [1]. The orientation of glass prepreg fabric layers plays a key role in determining the stiffness and strength of sandwich panels, which confirms the possibility of optimisation through appropriate laminate architecture [2]. Studies of CFRP composites have shown that single- and multi-layer systems differ in their response to tension and bending depending on the configuration of the layers and the direction of the load [3]. Epoxy–glass laminates with multi-axial fabric are characterised by a specific stress–strain curve during bending, which is strongly dependent on the orientation of the fibres and the layer arrangement [4]. The addition of carbon fibres or glass microspheres to the polymer matrix affects the flexural properties and fracture morphology, highlighting the importance of the type and distribution of the filler [5]. Studies of polymer/CNT nanocomposites have shown that the elongated shape and appropriate content of carbon nanotubes significantly improve flexural strength while maintaining ductility [6]. Hybrid laminates combining glass, carbon or aramid fibres allow a favourable compromise between stiffness and delamination resistance to be achieved with proper structural design [7].

In recent years, considerable attention has been paid to the use of carbon materials as fillers in polymer composites. In addition to traditional carbon sources such as graphite and carbon nanotubes, there is growing interest in sustainable and waste forms of carbon obtained through pyrolysis. Recent studies on the synthesis and modification of nanocarbons using microwave technology have shown that properly controlled process conditions can significantly influence the morphology, porosity and surface chemistry of carbon materials, which in turn improves their compatibility with polymer matrices and the mechanical properties of composites [8]. In this context, the carbonisate of MDF waste obtained through pyrolysis is an alternative, environmentally friendly source of functional carbon filler. Its use is in line with the concept of a circular economy and, at the same time, contributes to the improvement of selected mechanical properties of epoxy–glass laminates.

Furthermore, a comprehensive overview presented in Nanocarbon/Epoxy Composites: Preparation, Properties, and Applications highlights the diversity of methods for producing and functionalizing carbon fillers, as well as their incorporation into epoxy matrices to enhance mechanical, thermal and electrical properties [9]. It was emphasised that filler morphology, dispersion and surface modification are critical factors governing composite behaviour.

The dynamic development of the furniture and construction industries leads to the generation of increasing amounts of wood-based panel waste, including medium-density fibreboard (MDF). Disposing of these materials poses a significant environmental challenge because their composition, which includes synthetic resins and various chemical additives, makes mechanical recycling difficult. One of the most promising methods of managing this type of waste is pyrolysis, which allows for the recovery of gaseous, liquid and solid fractions. The solid fraction, carbonised material can be treated as a specific type of biocarbon derived from wood-based materials, generating increasing amounts of wood-based panel waste, including (MDF), which poses a significant environmental problem due to its disposal. The use of this by-product in polymer composites is in line with the concept of a circular economy, while also reducing material costs and improving certain mechanical properties of laminates [10]. Numerous studies have shown that biocarbon and its derivatives improve both the structural and functional properties of polymer composites. Incorporating biocarbon into epoxy matrices increases flexural strength, modulus of elasticity and surface hardness [11,12,13,14]. In a study on carbon fibre-reinforced bioepoxy composites, biocarbon was found to be an effective filler for increasing stiffness [11]. Other studies have shown that the addition of biocarbon to polymer matrices can increase the thermal resistance and dimensional stability of materials [14]. Interesting results were also obtained for nano-biocarbon obtained from olive waste—it improved the Young’s modulus and mechanical properties of epoxy composites [14]. In addition to biocarbon, the effect of other carbon fillers has also been extensively studied. The addition of carbon nanofibres (CNF) to epoxy resin significantly increased flexural strength and elastic modulus, with the effect depending on the content and dispersion of the filler [15]. Similar observations were made for epoxy composites modified with aluminium oxide nanopowders in the polymorphic γ-Al_2_O_3_ variety, where the beneficial mechanical effect was supplemented by an analysis of destruction processes using techniques combined with Kolmogorov–Sinai metric entropy ($E_{K-S}$) [16].

$E_{K-S}$ is a tool for analysing non-linear dynamics, measuring the degree of complexity and chaos of a process. In the context of strength testing of materials, it allows for the quantification of the rate at which the system (in the sense of a sample-strength testing machine pair) loses predictability, which enables the identification of critical moments occurring in the material during the test.

In the last few years, metric entropy ($E_{K-S}$) as a tool for assessing the dynamics of deformation processes has become increasingly popular. Published studies have shown that $E_{K-S}$ allows for the detection of critical points in both anisotropic materials [17] and metals [18]. The studies found that $E_{K-S}$ is useful in analysing the results of strength tests, as it allows the identification of stages of material degradation before maximum strength is reached [17,18]. Our own previous studies [19,20,21] confirm the observations found in the literature. It has been shown that the addition of 5–7.5% of 500 μm MDF carbonised material can significantly increase the hardness of epoxy–glass laminates [19], with the effect depending on the matrix/reinforcement ratio. In tensile tests, however, a decrease in strength was observed with a simultaneous increase in deformability [20]. In another study, the tensile test results showed a slight deterioration in strength associated with the presence of defects and discontinuities (pores, microcracks, local delamination) observed in the SEM analyses [21]. In these works, the Kolmogorov–Sinai metric entropy calculations were successfully applied to describe the dynamics of material damage and identify individual stages of structural transformations [21].

A summary of the existing literature reports and obtained results indicates that carbonised material (biocarbon) can be a valuable filler in epoxy composites, but its effect strongly depends on the type of fraction, by per cent fraction, matrix/reinforcement ratio and adhesion quality in the fibre–matrix–filler system. The research gap mainly concerns the determination of the range of processing parameters that allow a compromise between relatively high strength, hardness and deformability, as well as the integration of classical strength testing methods with modern analytical tools such as $E_{K-S}$.

This study focused on the flexural strength of epoxy–glass laminates with the addition of carbonisate obtained from the pyrolysis process of medium-density fibreboard (MDF).The tests were carried out on samples with different matrix/reinforcement ratios (60/40 and 65/35) and with the addition of 5% and 7.5% filler with a fraction up to 500 μm. The analysed parameters of flexural strength and deformability were also assessed using metric entropy calculations, which enable the identification of critical states during loading.

This article aims to extend previous research [19,20,21] with an analysis of the behaviour of laminates in a bending test, thus constituting a natural continuation of ongoing work on the influence of MDF carbonisate on the strength and impact properties of composites.

## 2. Materials and Methods

**Figure 1:**
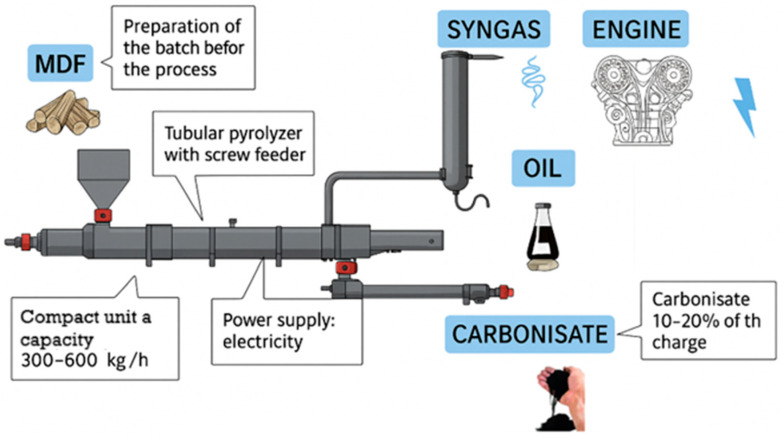
Schematic representation of the medium-density fibreboard (MDF) pyrolysis process.

The carbonisate employed in this study was obtained through the pyrolysis of medium-density fibreboard (MDF) waste, which involves the thermal decomposition of matter without access to air. The results of the thermal decomposition of substances in anaerobic conditions are the primary pyrolysis products, i.e., pyrolysis gas, liquid fraction (pyrolysis oil) and solid fraction (carbonised material) [22]. The pyrolysis process was carried out using a continuous flow pyrolysis reactor, as illustrated in Figure 1.

**Figure 2:**
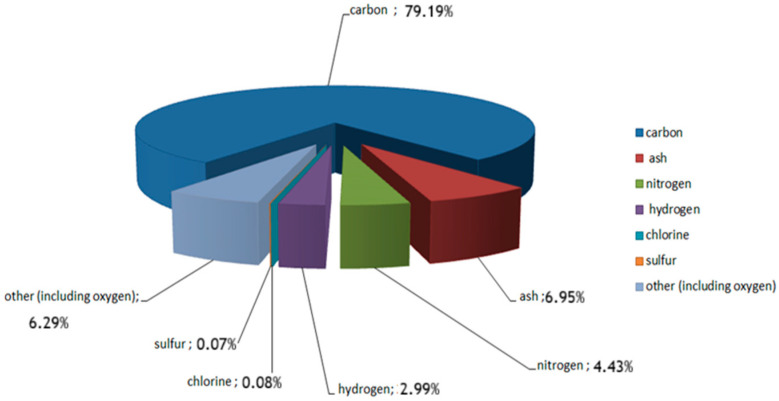
Chemical composition of the carbonisate obtained from medium-density fibreboard (MDF) furniture waste after pyrolysis.

Carbonisate obtained from furniture waste (including MDF boards) has been previously characterised in terms of chemical composition and morphology [19,21]. The chemical composition is shown in Figure 2.

**Figure 3:**
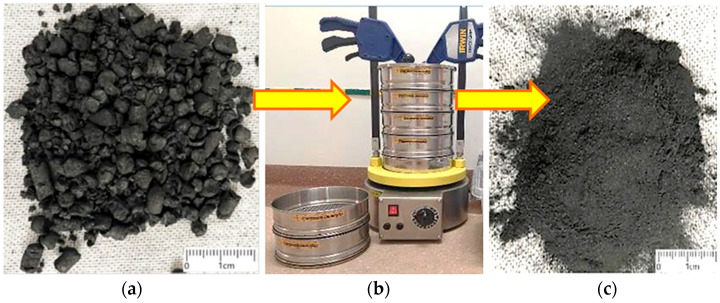
Carbonisate (**a**) after pyrolysis, (**b**) sieve shaker and (**c**) after crushing and screening to a 500 μm fraction.

To ensure repeatable mechanical properties of the composites, the filler particles were prepared to have the most uniform shape and controlled dimensions. The carbonised material was crushed and sieved on a LAB 11-200 sieve shaker (EKO-LAB, Jasień/Brzesko, Poland), obtaining a fraction with a particle size of up to 500 μm (Figure 3).

An EM 1002/450/125 emulsion mat (PHU Krisko, Lublin, Poland) with random orientation of E-glass fibres was used as reinforcement for the laminates, and Epidian 6 epoxy resin with Z-1 hardener (SARZYNA CHEMICAL sp. z o.o., Nowa Sarzyna, Poland) as the matrix, in accordance with the manufacturer’s recommendations. The properties of the materials (viscosity, density, epoxy number, by per cent per unit area, fibre diameter) are discussed in [19,20,21]. The filler was carbonised material with a fraction up to 500 μm in cross-section. A series of laminates was prepared for two nominal matrix/reinforcement ratios: 60/40 and 65/35, with the addition of 5% and 7.5% carbonisate (Table 1). The simplest method for manufacturing composite elements is hand lay-up. This process involves placing successive layers of glass mat in a mould and impregnating them with epoxy resin using rollers. The proportions of resin, mat and char required to fully saturate the reinforcement and obtain a material with optimal mechanical properties were then determined. To ensure a uniform dispersion of the carbonisate within the epoxy matrix, the filler was slowly added to the resin under continuous mechanical stirring for 3–5 min at low speed (below 200 rpm), followed by manual mixing to eliminate possible agglomerates and ensure homogeneity of the mixture before impregnation.

A series of laminates was produced for two nominal matrix-to-reinforcement ratios: 60/40 (series A, A1, A2) and 65/35 (series B, B1, B2), modified with 5% and 7.5% of carbonisate (with a grain size of up to 500 μm). Each composite consisted of 10 layers of glass mat. The reference samples (A and B) were unmodified epoxy–glass laminates, while the modified samples contained 5% (A1, B1) or 7.5% (A2, B2) of char, respectively.

For each laminate type, 10 samples were prepared for mechanical testing to ensure statistical reliability of the results.

## 3. Results

**Figure 4:**
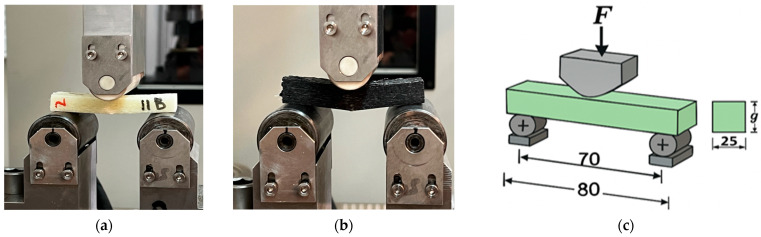
Sample from series B (**a**), series with added carbonisate B1 (**b**), (**c**) geometry and dimensions of samples for three-point bending test (g—sample thickness).

The laminates were made using the manual impregnation method. Samples for three-point bending tests were cut from pre-cured boards using a water jet cutting method and prepared in accordance with PN-EN ISO 14125:2001 [23]. The samples were subjected to three-point bending tests on a ZwickRoell MPMD P10B hydraulic testing machine controlled by TestXpert II software (version 3.6.1; ZwickRoell Group, Ulm, Germany). The load was applied by a movable upper support, and the force and deformation were recorded by the machine’s sensors. Data recording and pre-processing were performed in TestXpert II. The test was carried out in accordance with PN-EN ISO 14125:2001 [23]. The geometry and dimensions of the samples are shown in Figure 4.

Ten three-point bending tests were performed for each combination. The average values of flexural strength $\sigma_{fM}$ and flexural strain $\varepsilon_{fM}$ are listed in Table 2.

**Figure 5:**
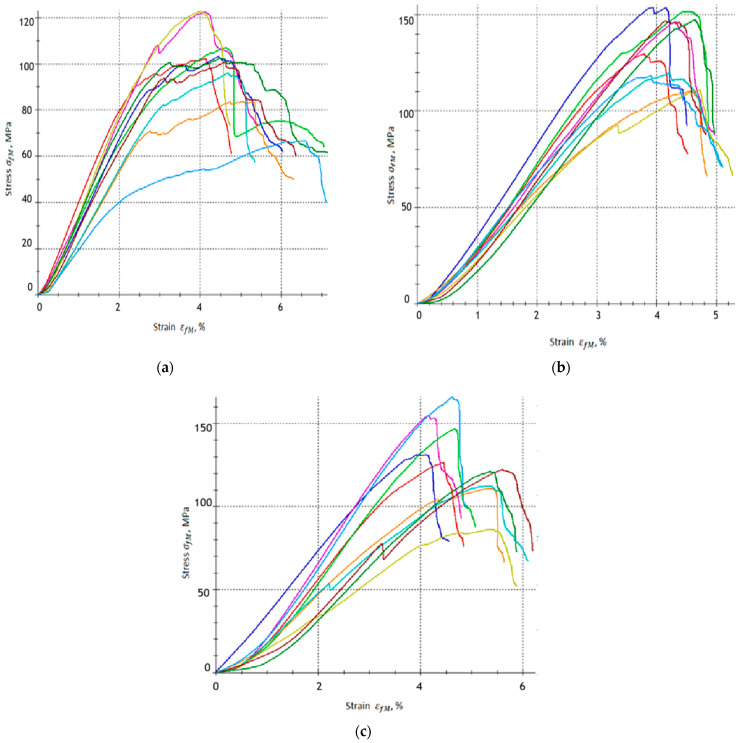
Stress–strain relationships obtained from the three-point bending test for epoxy–glass composites: (**a**) Group A, (**b**) group A1 and (**c**) group A2.

Figure 5 shows the stress–strain curves in three-point bending for each variant of the tested material, A, A1 and A2, respectively.

**Figure 6:**
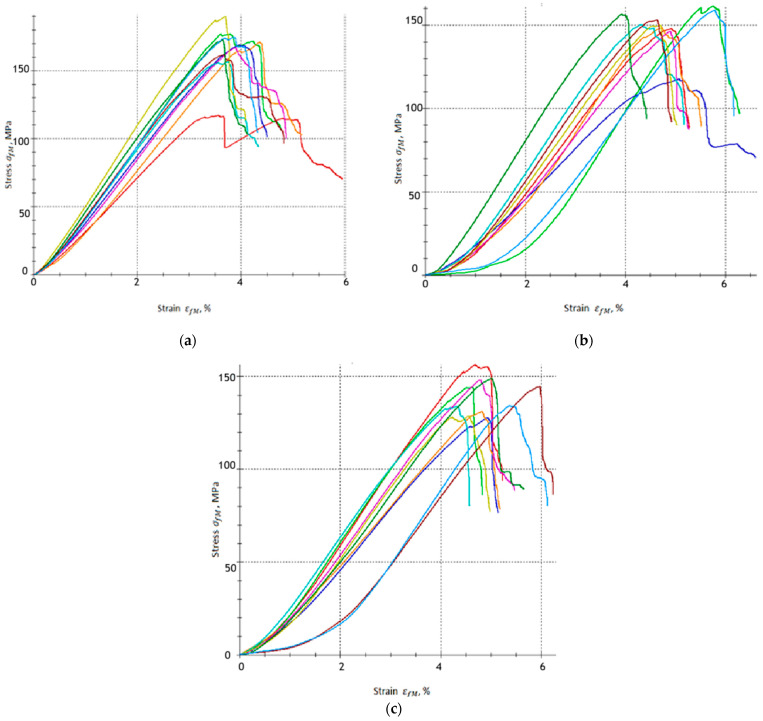
Stress–strain relationships obtained from the three-point bending test for epoxy–glass composites: (**a**) Group B, (**b**) group B1 and (**c**) group B2.

Figure 6 shows the stress–strain curves in three-point bending for each variant of the tested material, B, B1 and B2, respectively.

Based on the obtained results, it can be concluded that the addition of a carbonisation agent influences the mechanical response of the tested composites, especially in terms of bending strength ($\sigma_{fM}$) and flexural strain ($\varepsilon_{fM}$). The reference samples without carbonisate (0% by wt.), groups A and B, exhibited flexural strength $\sigma_{fM}$ = 100.56 MPa and $\sigma_{fM}$ = 165.45 MPa, respectively. The flexural strain for these samples is $\varepsilon_{fM}$ = 4.634% and $\varepsilon_{fM}$ = 3.792%, respectively. These results confirm the stable mechanical behaviour of the base laminates, with the differences in flexural strength and flexural strain resulting mainly from different resin-to-reinforcement ratios, i.e., 60/40 for group A and 65/35 for group B.

The addition of 5% carbonisate < 500 μm (by per cent, samples from group A1) results in an increase in flexural strength to $\sigma_{fM}$ = 133.30 MPa, which means an increase in flexural strength of approximately 32.56%. The deformation decreased slightly to $\varepsilon_{fM}$ = 4.269%, which indicates that in this amount the carbonisate stiffened the matrix and increased its brittleness, limiting the material’s ability to deform. The increase in strength is probably because the carbonisate particles were well dispersed in the matrix, stiffening it and improving the effective transfer of stresses to the glass fibres without critically reducing the ability to deform.

Samples from group A2 (7.5% carbonisate additive by per cent) also show an increase in flexural strength, reaching values of $\sigma_{fM}$ = 127.79 MPa, which corresponds to an increase of approximately 27.08% compared to the base samples from group A (without carbon-based filler). The flexural strain for group A2 was increased to $\varepsilon_{fM}$ = 4.887%, which represents a slight increase of approximately 0.055% compared to the A group samples.

A different effect was observed in the case of series B samples. The base variant group B (without additive) is characterised by the highest flexural strength among all samples ($\sigma_{fM}$ = 165.45 MPa) and deformation $\varepsilon_{fM}$ = 3.792%. The addition of 5% 500 μm of carbonisate (group B1 samples) resulted in a decrease in strength to $\sigma_{fM}$ = 149.05 MPa, which is a decrease of approximately 9.89% compared to the group B samples. At the same time, the deformation increased to $\varepsilon_{fM}$ = 4.867%, which indicates a greater ability of the sample to deform despite the reduction in load-bearing capacity. In the case of group B2 samples (7.5% carbonisate), the flexural strength was further reduced to $\sigma_{fM}$ = 139.76 MPa, i.e., by approximately 15.53% compared to variant B, while the deformation reached a value of $\varepsilon_{fM}$ = 4.908%. The results obtained suggest that in series B, the addition of carbonisate does not improve the strength of the composite but even reduces it. This may be related to the higher proportion of resin in relation to fibres, which, in combination with carbonate particles, leads to the formation of local defects and earlier initiation of cracks. At the same time, carbonisate particles may facilitate energy dissipation during bending, which explains the observed increase in the deformation $\varepsilon_{fM}$.

The results of series A indicate that the addition of carbonisate contributes to an increase in flexural strength while maintaining or even slightly increasing deformability, which can be associated with beneficial reinforcement of the matrix and improved stress transfer to the fibres. In series B, on the other hand, the addition of carbonisate reduction in flexural strength was accompanied by a moderate rise in strain. This suggests that in systems with a higher resin content, carbonisate particles may act as crack-initiating defects, reducing the load-bearing capacity of the material but at the same time increasing its deformation capacity.

### 3.1. Method of Calculation $E_{K-S}$

The Kolmogorov–Sinai metric entropy $E_{K-S}$ was calculated for sets of recorded flexural strain values $\varepsilon_{fM}$ obtained during three-point bending tests of samples from groups A, A1, A2, B, B1 and B2. The method of performing $E_{K-S}$ calculations on the sets of deformations $\varepsilon_{fM}$ did not differ significantly from the $E_{K-S}$ calculation method presented and described in detail in [21]. The calculations were based on the formula [24,25], which is an adaptation of Shannon’s entropy for discrete probability distributions.

(1) $$E_{K-S}=-\sum_{i=1}^{N}p_{i}\ln p_{i}.$$

where:

*p*—probability, *i*—of this state.

**Figure 7:**
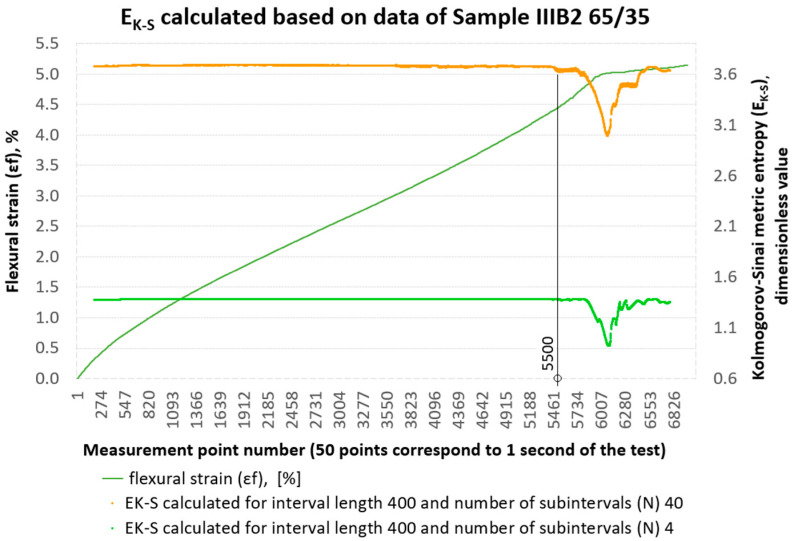
Results of metric entropy calculations performed on the deformation set $\varepsilon_{fM}$ of sample No. IIIB2 for the assumed interval length of 400 and 4 and 40 subintervals, respectively.

The calculation process itself was performed using the proprietary programme ‘Entropy K_S’ ver. 1.20 [21]. At this point, it is worth explaining how the initial parameters, i.e., the assumed interval length and the number of subintervals assumed for this interval—*N* from Formula (1), affect the obtained $E_{K-S}$ calculation results. To illustrate the procedure of the $E_{K-S}$ analysis, calculations were carried out for one of the representative specimens from group B2. On the set of deformations $\varepsilon_{fM}$ of the sample marked as IIIB2 $E_{K-S}$ calculations were performed for an interval with a length of 400 measurement points and 4 subintervals and 40 subintervals, respectively. The results of the calculations are shown in Figure 7. In this figure, we can see that the initial value of $E_{K-S}$ changes depending on the number of subintervals used. Increasing the number of subintervals increases the initial value of the metric entropy. In Figure 7, both $E_{K-S}$ curves (orange and green) show no visible fluctuations until approximately measurement point no. 5500. This is the value at which material damage is visible to the naked eye. The use of such a long interval (400 measurement points) for $E_{K-S}$ calculations does not add anything new to what we obtain from the strength testing machine itself in a three-point bending test, i.e., the values $\sigma_{fM}$ and $\varepsilon_{fM}$ (flexural strength and flexural strain). Even with varying numbers of subintervals, from 4 to 40, the method remains insufficiently sensitive to detect minor internal changes in the structure of the loaded composite.

**Figure 8:**
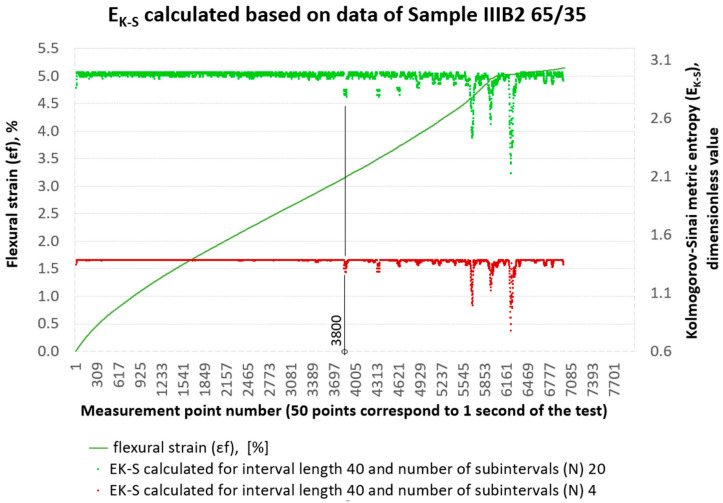
Results of metric entropy calculations performed on the deformation set $\varepsilon_{fM}$ of sample No. IIIB2 for the assumed interval length of 40 and 4 and 20 subintervals, respectively.

For the same data set, Kolmogorov–Sinai entropy calculations were then performed, assuming an interval length of 40 and dividing it into 4 and 20 subintervals, respectively. The results of the $E_{K-S}$ calculations are shown in Figure 8. With these initial parameters, we can already capture the qualitative changes occurring inside the material from measurement point No. 3800. From the course of the $E_{K-S}$ curves in Figure 8, we can clearly see that reducing the interval length to 40 measurement points from the previous 400 increases the ‘resolution’ of the $E_{K-S}$ tool. This is consistent with the principles of time series analysis, where a smaller time interval (in this case, the length of the adopted measurement interval) allows for the detection of more detailed, local changes in the signal, while a longer interval averages these changes, leading to a more general picture of the dynamics of the system [24]. A larger number of subintervals in Figure 8, i.e., 20, creates a more detailed division of the phase space, which increases the amount of output information. This, in turn, translates into a higher initial entropy value. A smaller number of subintervals, i.e., 4, gives a more general division, resulting in a lower base value of $E_{K-S}$. By increasing the number of subintervals, we are effectively ‘looking’ at the data with greater precision, which allows us to capture more subtle, chaotic fluctuations in the system. This more detailed picture of the dynamics is then quantified by a higher entropy value $E_{K-S}$, as can be seen in the graph, where 20 subintervals in the same 40 intervals give a base value of the entropy metric of around 3.0, and 4 subintervals give a value of around 1.4.

Therefore, the more precisely we analyse the dynamics of the system (the more subdivisions we introduce), the more chaos we can perceive, and the base value $E_{K-S}$ reflects this ‘resolution’. $E_{K-S}$ which is the amount of entropy per unit of time, does not have a constant base value [26]. The initial parameters are like the focal length of a lens used for observation, which we impose on ourselves.

- By selecting a larger number of subdivisions, such as 20 subdivisions compared to 4, we increase the resolution, which leads to a higher base value of $E_{K-S}$ (approx. 3.0 vs. 1.4). This greater ‘zoom’ reveals more subtle, chaotic fluctuations that are ignored at lower resolutions.
- In turn, by reducing the length of the interval (from 400 to 40), we narrow the observation window, which allows us to see a significantly larger number of rapid and frequent fluctuations in $E_{K-S}$, which translates into higher ‘nervousness’ of the obtained signal.

Ultimately, the base value $E_{K-S}$ reflects our choice of scale—how precisely we want to describe the chaos of the system, and not just its state. Sudden and intense variability (fluctuation) of $E_{K-S}$ is a key indicator of impending instability in the structure of the material. At this point, it seems reasonable to introduce an additional tool to verify the correctness of the scale selection, and thus a tool to verify the adopted interval lengths and number of subintervals.

From Pensin’s theorem, we know that $E_{K-S}$ equals the sum of positive Lyapunov exponents [27]:

(2) $$E_{K-S}\;=\;h_{\mu }\left(f\right)\;=\;\int \Sigma_{\lambda_{i}>0}{\;\lambda }_{i}d\mu \;$$

where:

*λ_i_*—positive Lyapunov exponents, and *μ* is an *f*-invariant measure.

Metric entropy is a measure of the unpredictability of a dynamic system. It tells us how much information is generated per unit of time. In our case, the dynamic system is a composite sample—a strength testing machine bending this sample under an increasing load (the test was conducted at a constant rate of force increase). Lyapunov exponents describe the rate at which trajectories diverge (or converge) along different directions in the phase space. If a positive Lyapunov exponent appears, the system exhibits chaotic sensitivity in that direction. Pensin argues that all the unpredictability of the system comes from stretching in the direction of positive Lyapunov exponents. We also know that phase portraits visualise trajectories in the phase space as follows:

for *λ* max < 0 → all trajectories land at a stable point;
for *λ* max = 0 → quasi-periodic trajectories appear (e.g., closed curves, tori);
for *λ* max > 0 → the phase image becomes ‘jagged’, chaotic attractors appear.

**Figure 9:**
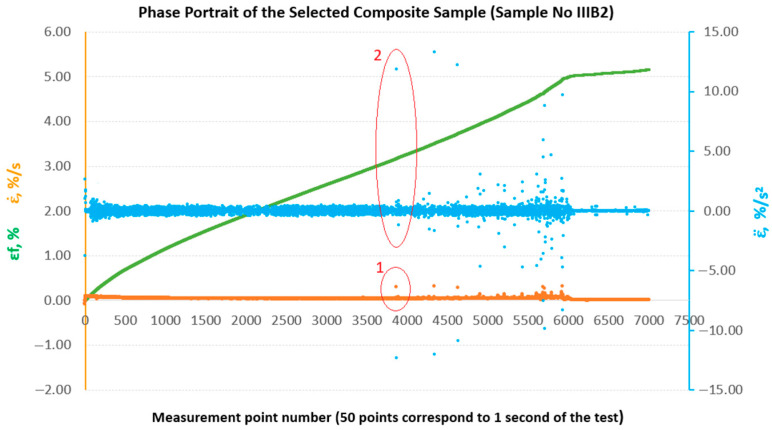
Phase portraits $\dot{\varepsilon }$ and $\ddot{\varepsilon }$ made from the set of deformations of sample No. IIIB2.

In the past, attempts have been made to reconstruct the phase space for the results of strength tests of composite materials in comparison with the results of metric entropy calculations $E_{K-S}$ [28]. Figure 9 shows phase portraits obtained from the set of strains $\varepsilon_{fM}$ of the sample analysed above, No. IIIB2. In our case, the phase portraits represent, respectively, $\dot{\varepsilon }$—strain rate and $\ddot{\varepsilon }$—strain acceleration, which are the first and second derivatives of strain with respect to the measurement sequence, which, in practice, can be treated as a time-dependent function (50 consecutive measurement points correspond to 1 s of testing). In Figure 9, the phase portraits $\dot{\varepsilon }$ and $\ddot{\varepsilon }$ begin to become clearly jagged (areas 1 and 2 marked in red in Figure 9) from the measurement point around number 3800, which coincides with a local decrease in the value of $E_{K-S}$ calculated for an interval of 40 measurement points (Figure 8).

For further analysis of the strength properties of all tested samples, it was decided to use calculations for the same interval length, i.e., 40, and 4 sub-intervals.

**Figure 10:**
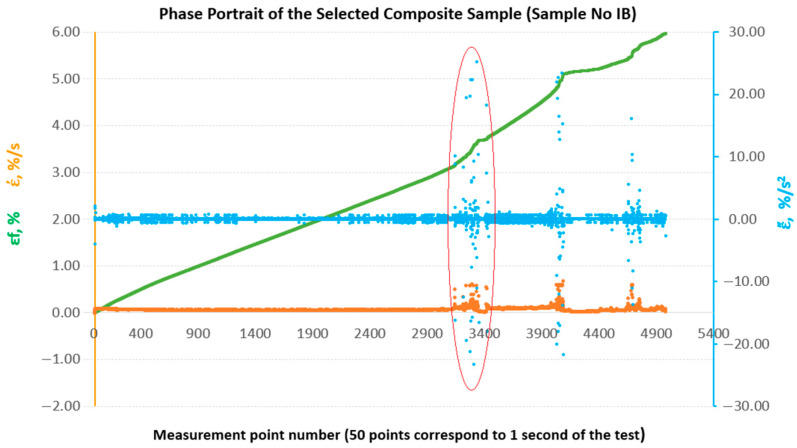
Phase portraits $\dot{\varepsilon }$ and $\ddot{\varepsilon }$ made from the set of deformations of sample No. IB.

**Figure 11:**
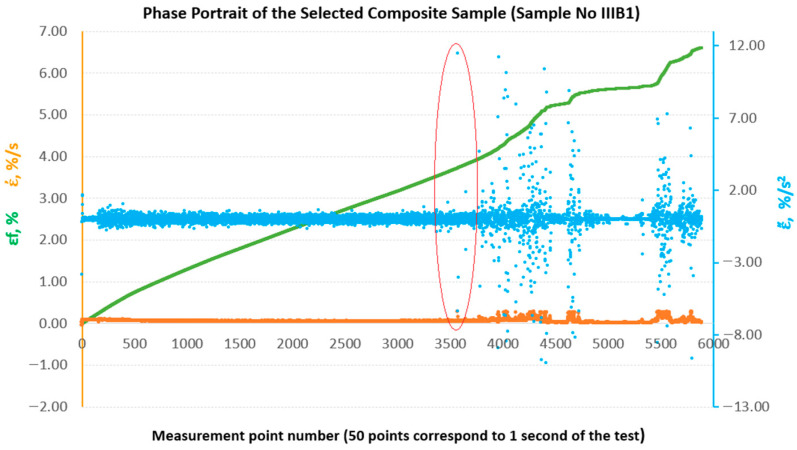
Phase portraits $\dot{\varepsilon }$ and $\ddot{\varepsilon }$ made from the set of deformations of sample No. IIIB1.

**Figure 12:**
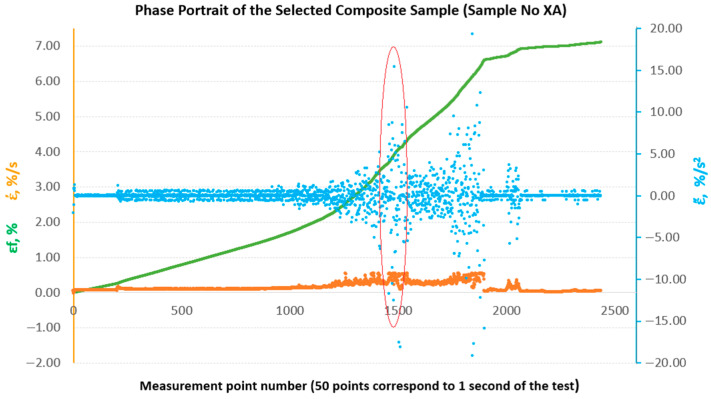
Phase portraits $\dot{\varepsilon }$ and $\ddot{\varepsilon }$ made from the set of deformations of sample No. XA.

**Figure 13:**
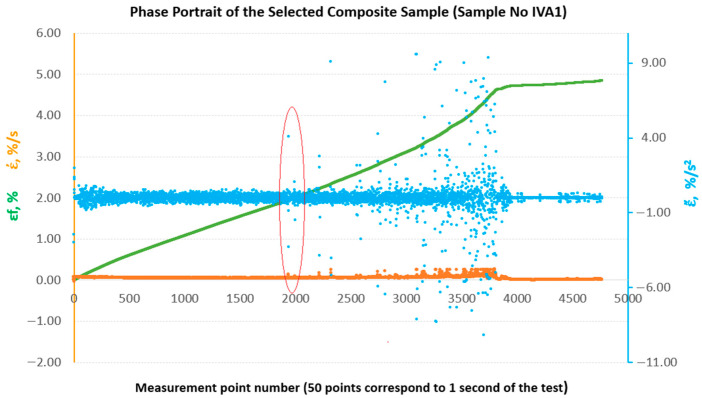
Phase portraits $\dot{\varepsilon }$ and $\ddot{\varepsilon }$ made from the set of deformations of sample No. IVA1.

**Figure 14:**
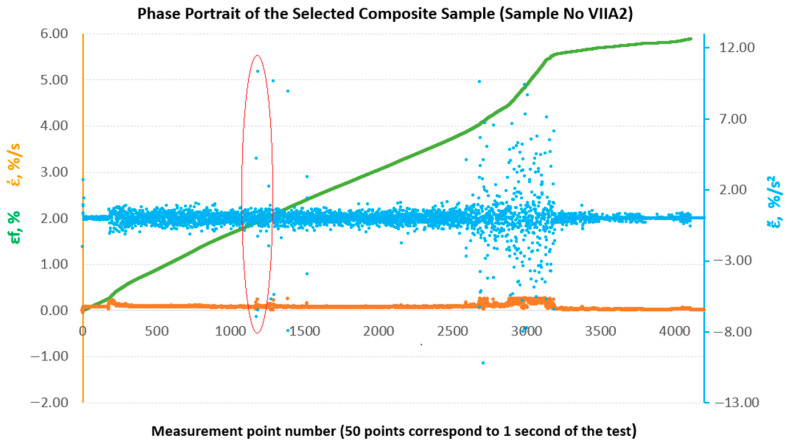
Phase portraits $\dot{\varepsilon }$ and $\ddot{\varepsilon }$ made from the set of deformations of sample No. VIIA2.

To enable a comparative evaluation of the deformation dynamics between the analysed laminates, phase portraits ($\dot{\varepsilon }$-$\ddot{\varepsilon }$) were generated for all sample groups (B, B1, A, A1, A2), Figure 10, Figure 11, Figure 12, Figure 13 and Figure 14.

### 3.2. Evaluation of Strength Properties Using Metric Entropy Analysis

**Figure 15:**
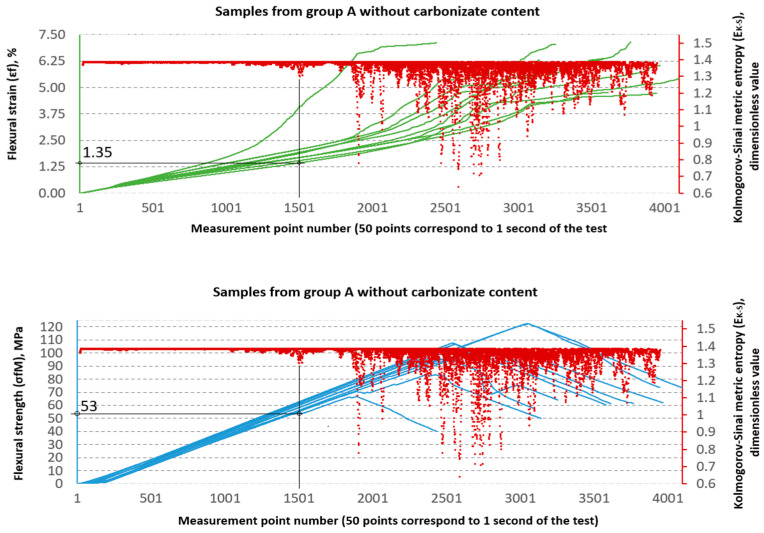
Designation $\varepsilon_{fK-S}$ and $\sigma_{fMK-S}$ for samples from group A (60/40, 0%—carbonisate).

**Figure 16:**
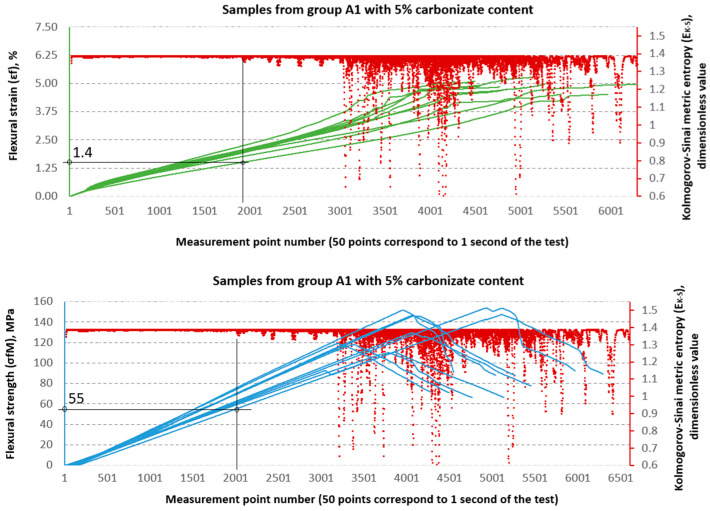
Designation $\varepsilon_{fK-S}$ and $\sigma_{fMK-S}$ for samples from group A1 (60/40, 5%—carbonisate).

**Figure 17:**
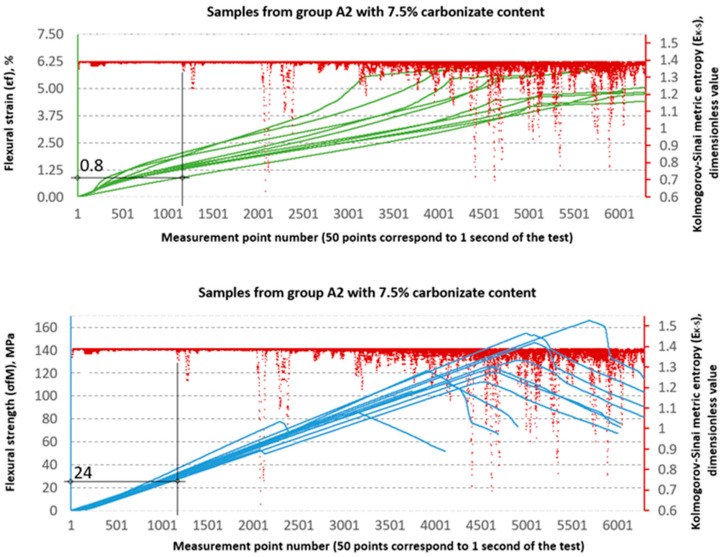
Designation $\varepsilon_{fK-S}$ and $\sigma_{fMK-S}$ for samples from group A2 (60/40, 7.5%—carbonisate).

**Figure 18:**
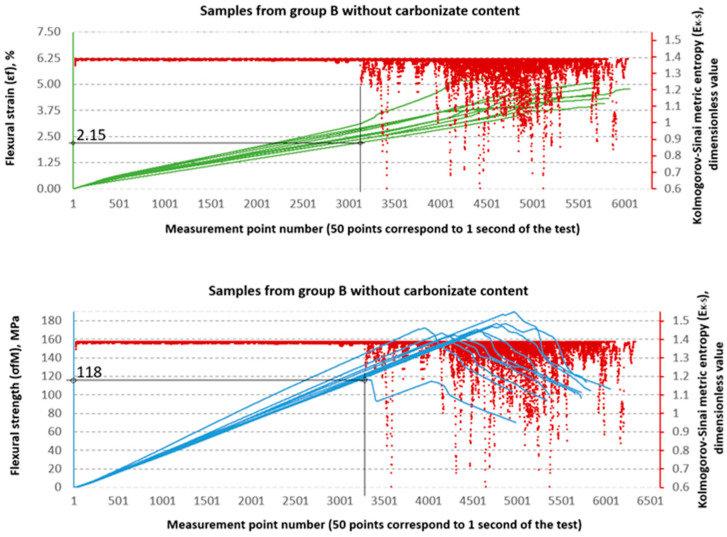
Designation $\varepsilon_{fK-S}$ and $\sigma_{fMK-S}$ for samples from group B (65/35, 0%—carbonisate).

**Figure 19:**
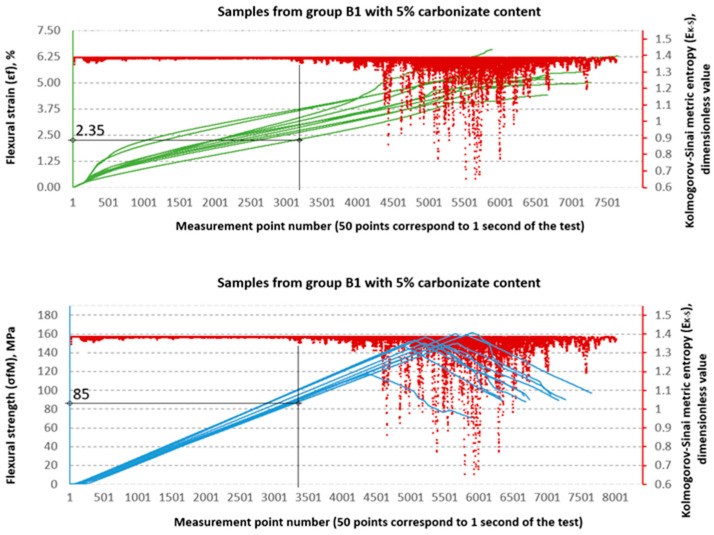
Designation $\varepsilon_{fK-S}$ and $\sigma_{fMK-S}$ for samples from group B1 (65/35, 5%—carbonisate).

**Figure 20:**
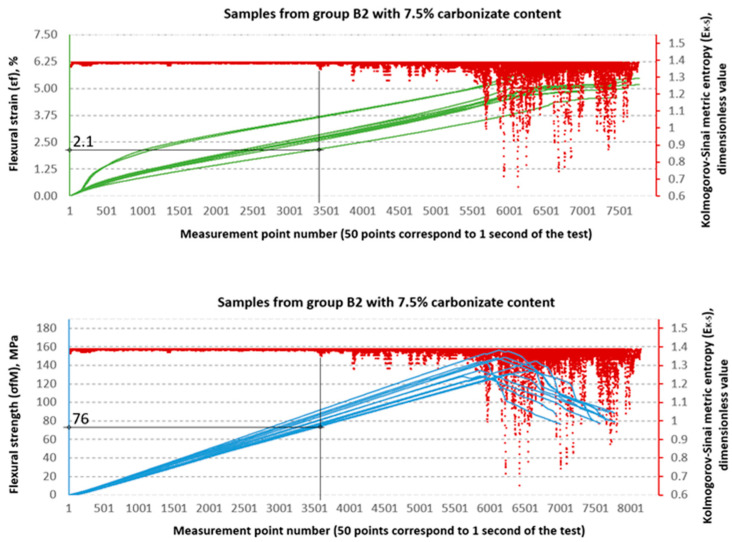
Designation $\varepsilon_{fK-S}$ and $\sigma_{fMK-S}$ for samples from group B2 (65/35, 7.5%—carbonisate).

The Kolmogorov–Sinai metric entropy approach method was used to assess the durability of epoxy–glass composites with the addition of carbonisate. According to the authors, this method allows for a relatively simple determination of the safety factors necessary for the design of bent structures made of this material. The six consecutive figures below, i.e., Figure 15, Figure 16, Figure 17, Figure 18, Figure 19 and Figure 20, present a summary of the calculation results for all six groups of tested materials, i.e., A, A1, A2, B, B1 and B2. Each of the figures below consists of two parts. The upper part of the figures shows the deformation curves $\varepsilon_{fM}$ (green) as a function of successive measurement points, i.e., as a function of time. The lower part of the figures show the stress curves $\sigma_{fM}$ (blue), also as a function of successive measurement points in the test. Both parts of the graphs show the summary results of the $E_{K-S}$ calculations. The graphical summary of the $E_{K-S}$ calculation results take the form of a scatter plot ($E_{K-S}$, 1 value point corresponds to 40 points of the interval for which this value was calculated) [21]. In Figure 15, Figure 16, Figure 17, Figure 18, Figure 19 and Figure 20, the smallest values obtained for each group of materials, expressed as the % deformation and stress values expressed in MPa, at which significant qualitative changes in the material structure occur, are marked with black lines and described on the axes. These values are denoted as: $\varepsilon_{fK-S}$ and $\sigma_{fMK-S}$, respectively. The values were verified each time by creating phase portraits $\dot{\varepsilon }$ and $\ddot{\varepsilon }$, as described in Figure 9.

The graphical analysis shows that series A is characterised by greater strength gain at lower structural stability thresholds. In contrast, series B is characterised by lower strength but higher resistance to local chaotic changes. This confirms the conclusions of classical strength tests and demonstrates the added value of using metric entropy as a diagnostic tool.

Table 3 presents a comparison between the mean values of stressσ and strain ε obtained from the static three-point bending test and the corresponding parameters—relative strain $\varepsilon_{K-S}$ and stress $\sigma_{K-S}$ derived from the Kolmogorov–Sinai metric entropy analysis $E_{K-S}$.

An analysis of the threshold values presented in Table 3 shows clear differences between series A and B. In series A samples, the $\sigma_{fMK-S}$ values are significantly lower than the destructive strength. For example, in sample A, the structural stability limit is 53 MPa, which is only 52.7% of the maximum value (100.56 MPa). This is even more evident in sample A2, where $\sigma_{fMK-S}$ reaches only 24 MPa, or 18.8% of the destructive value (127.79 MPa). At the same time, the $\varepsilon_{fK-S}$ values remain relatively low (0.8–1.4%), which indicates that these materials, despite their relatively high flexural strength obtained from the testing machine software as $\sigma_{fM}$ and $\varepsilon_{t}$ values, in fact undergo significant qualitative changes in their structure much earlier, as shown by the $\sigma_{fMK-S}$ and $\varepsilon_{fK-S}$ values, respectively.

In the B series samples, the situation is different but analogous to the results obtained from the strength testing machine software. The base variant B achieves $\sigma_{fMK-S}$ = 118 MPa, which is as much as 71.3% of the maximum strength (165.45 MPa). Similar relationships are observed in modified samples—in the case of B1, the threshold value of 85 MPa is 57.0% of the destructive value, and for B2, 76 MPa, i.e., 54.4% of the final value. The $\varepsilon_{fK-S}$ values in this series are higher (2.1–2.35%), which indicates a greater ability to deform before reaching the destruction threshold. These data suggest that the B series is characterised by lower maximum strength but greater stability throughout the loading process.

**Figure 21:**
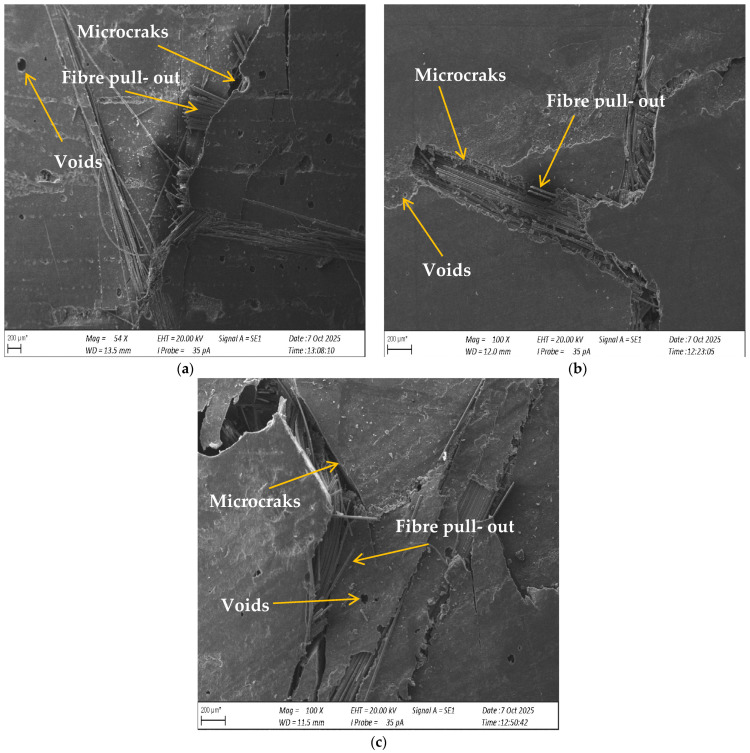
Scanning Electron Microscope (SEM) micrograph of the fracture surface of laminate (**a**) group B, (**b**)group B1 and (**c**) group B2.

To complement the mechanical test results and gain a deeper insight into the failure mechanisms of the composites, a SEM analysis of the fracture surfaces was performed. Figure 21 presents Scanning Electron Microscope (SEM) micrographs showing the fracture surfaces of laminates with a 65/35 matrix-to-reinforcement ratio: (a) reference sample B, (b) laminate B1 with 5% carbonisate and (c) laminate B2 with 7.5% carbonisate.

Reference laminate (a) exhibits a relatively uniform surface with well-embedded glass fibres and limited fibre pullout, indicating good adhesion between the matrix and reinforcement. In contrast, laminate B1 (b) exhibits voids, local microcracks and partial fibre debonding, suggesting that the addition of 5% char slightly reduces interfacial adhesion. The most pronounced structural degradation is seen in laminate B2 (c), where larger cracks, voids and extensive fibre pullout can be observed. These microstructural features correlate with the decrease in flexural strength observed for the 65/35 composites, indicating that excessive filler content can cause defect formation that acts as a crack initiation site.

## 4. Discussion

The conducted research enabled an in-depth analysis of how the impact of carbonisate affects the behaviour and strength characteristics of epoxy–glass composites and an assessment of the dynamics of their damage processes. The interpretation of the obtained results indicates complex mechanisms of interaction between the filler and the epoxy matrix, which are strongly dependent on the composition of the composite.

In series A (60/40), the addition of carbonisate brought a measurable benefit in the form of increased flexural strength. The introduction of 5% filler (samples from group A1) increased strength by 32.56%, and 7.5% (group A2 samples) by 27.08% compared to the reference material. This increase can be attributed to the effective stiffening of the matrix by well-dispersed carbonisate particles, which improved the effective transfer of stresses to the glass fibres. Interestingly, an increase in the plasticity of the material was also observed in the samples of group A2, suggesting that at higher filler content, it could have an additional function of inhibiting the development of microcracks in the matrix.

A completely different situation was observed in sample group B (65/35), where a higher resin content in the composition led to a negative effect of the carbonisate additive. The flexural strength decreased by 9.89% for samples in group B1 (5% C) and by 15.53% for samples in group B2 (7.5% C). This can be explained by the fact that in a matrix with a larger volume, the carbonisate particles, instead of strengthening it, began to act as local defects and stress concentrators, initiating premature cracking of the material. The change in the failure mechanism is confirmed by the simultaneous, marked increase in the deformation in both modified samples of group B. This suggests that the presence of particles facilitated energy dissipation during bending, which translated into greater plasticity, but at the expense of flexural strength.

A key aspect of this study was the application of the Kolmogorov–Sinai entropy metric ($E_{K-S}$) approach to analyse the strength behaviour properties, which has made it possible to go beyond standard strength assessment. This method made it possible to precisely indicate the threshold beyond which irreversible structural changes occur in the material. The determined limit values for stress ($\sigma_{fMK\_S}$) and strain ($\varepsilon_{fMK\_S}$) are significantly lower than the maximum destructive values obtained from the machine software. For example, for the base samples of group B, the maximum strength was $\sigma_{fM}$ = 165.45 MPa, while the damage initiation threshold was already determined at $\sigma_{fMK\_S}$ = 118 MPa. The case of sample A2 is particularly interesting, where, despite an increase in ultimate strength ($\sigma_{fM}$ = 127.79 MPa), the structural stability threshold dropped dramatically to $\sigma_{fMK\_S}$ = 24 MPa. This means that although this material is capable of transferring high bending loads, degradation processes begin very early on. This confirms the thesis that metric entropy is a highly sensitive tool capable of detecting subtle changes in the dynamics of a system that are invisible on a standard stress–strain curve.

The classic approach to determining safety factors (SF) in composite material designs is based on maximum destructive values $\sigma_{fMK-S}$ and $\varepsilon_{fK-S}$. In this approach, permissible stresses are defined as an appropriate fraction of the destructive values, depending on the adopted safety factor. However, the results obtained in this study show that in the case of composites with carbonisate additives, the material degradation process begins much earlier than at the point of maximum strength.

The application of the metric entropy method, therefore, provides a new perspective on determining safety factors in composite materials. Instead of referring working loads exclusively to destructive values, it is more reasonable to adopt the thresholds $\sigma_{fMK-S}$ and $\varepsilon_{fK-S}$ as the basis for determining permissible stresses. In this approach, the safety factor better reflects the actual durability of the material, taking into account the moment of degradation initiation, rather than only its final failure.

## 5. Conclusions

Based on the conducted research and analysis of the results, the following conclusions were formulated:

The influence of the carbonisate filler on the strength behaviour of epoxy–glass laminates shows a dual nature, which is determined by the matrix-to-reinforcement ratio. In composites with a higher reinforcement content (series A), the addition of the carbonisate increases flexural strength by up to 32.56%. In composites with a higher resin content (series B), the same addition reduces strength by up to 15.53%. In composites with a higher resin content (series B), the addition of carbonisate, despite reducing strength, increases the material’s deformation capacity, which indicates a change in the fracture mechanism. Analysis using Kolmogorov–Sinai metric entropy ($E_{K-S}$) calculations is an effective tool for identifying the early stages of composite structure degradation. Rapid fluctuations in $E_{K-S}$ values signal the onset of irreversible changes in the material that precede its macroscopic destruction. The method used allowed for the objective determination of the limit values of stresses ($\sigma_{fMK\_S}$) and strains ($\varepsilon_{fMK\_S}$), which define the actual threshold of the material’s permanent strength. These values are crucial for determining safe operating conditions and safety factors. The combination of standard mechanical testing with non-linear dynamics analysis is an advanced research approach that provides comprehensive knowledge about the behaviour of composite materials under load and allows their composition to be optimised for specific engineering applications.

## Figures and Tables

**Table 1:** Composition of epoxy–glass laminates prepared using the hand lay-up technique.

No.	Sample Code	Number of Samples Tested	Carbonisate Fraction [μm]	Number of Mat Layers	Resin Content %	Glass Mat Content %	Carbonisate Content %
1	A	10	-	10	60	40	0
2	A1	10	<500	10	60	35	5
3	A2	10	<500	10	60	32.5	7.5
4	B	10	-	10	65	35	0
5	B1	10	<500	10	65	30	5
6	B2	10	<500	10	65	27.5	7.5

**Table 2:** Mean results of the static three-point bending stress-test and strain values for samples containing various carbonisate contents and particle sizes. ↑ indicates an increase and ↓ a decrease relative to the reference sample.

No.	Sample Code	Carbonisate Fraction [μm]	Carbonisate Content %	$\sigma_{fM}$ Stress MPa	Difference Percentage %	$\varepsilon_{fM}$ % Strain	Difference Percentage %
1	A	-	0	100.56		4.634	
2	A1	500	5	133.30	32.56 ↑	4.269	0.079 ↓
3	A2	500	7.5	127.79	27.08 ↑	4.887	0.055 ↑
4	B	-	0	165.45		3.792	
5	B1	500	5	149.05	9.89 ↓	4.867	0.283 ↑
6	B2	500	7.5	139.76	15.53 ↓	4.908	0.294 ↑

**Table 3:** Summary of average strength parameters from the three-point bending tests and the corresponding values obtained through metric entropy evaluation.

Sample Code	$\varepsilon_{fM}$, %	$\varepsilon_{K-S}$, %	Changing the $\varepsilon_{K-S}$ Relative to $\varepsilon_{fM}$ %	$\sigma_{fM}$ MPa	$\sigma_{fMK-S}$ MPa	Change in $\sigma_{K-S}$ Relative to $\sigma_{fM}$ %
A	4.634	1.35	71	100.56	53	47
A1	4.269	1.40	67	133.30	55	59
A2	4.887	0.80	84	127.79	24	81
B	3.792	2.15	43	165.45	118	29
B1	4.867	2.35	52	149.05	85	43
B2	4.908	2.1	57	139.76	76	46

## Data Availability

The raw data supporting the conclusions of this article will be made available by the authors on request.
